# Evaluation of Antioxidant, Cytotoxic, and Anticancer Activities of Methanol Extract of *Annona reticulata* Linn. Leaves

**DOI:** 10.1155/sci5/5166065

**Published:** 2025-08-28

**Authors:** Tasnia Binte Bari Kabbo, Fahim Shahrier Rahman, Md. Sohel Rana, Pritesh Ranjan Dash

**Affiliations:** ^1^Department of Pharmacy, Jahangirnagar University, Savar, Dhaka, Bangladesh; ^2^Department of Pharmacy, Primeasia University, Banani, Dhaka, Bangladesh

**Keywords:** *Annona reticulate* Linn., anticancer, antioxidant, aqueous, cytotoxic activities

## Abstract

As potent therapeutic agents, the pharmacological potentials of natural substances have been the subject of recent research. Around the world, numerous tribes and ethnic communities have long used *Annona reticulata* Linn. (Family: *Annonaceae*) to treat variety of illnesses. Diverse therapeutic effects, including anthelmintic, antipyretic, antihyperglycemic, antiulcer, and antinociceptive properties are demonstrated by the plant. The current study assessed the plant's antioxidant, cytotoxic, and anticancer activities along with identifying probable responsible compounds for these effects via GC-MS analysis. The antioxidant potential was assessed using six assays. In DPPH and nitric oxide radical scavenging tests, good antioxidant property was demonstrated by test fraction with observed IC_50_ values of 83.72 and 107.92 μg/mL. The extract's reducing potential was found to enhance with enhancing concentration. The 139.5 ± 7.21 mg ascorbic acid equivalent/g extract was obtained as the total antioxidant capacity value. The calculated total phenolic and flavonoid contents values for the extract were 69.73 ± 0.26 mg gallic acid equivalent/g extract and 93.62 ± 0.15 mg quercetin equivalent/g extract. The extract showed promising cytotoxic property in the brine shrimp lethality bioassay. In this study, the observed LC_50_ value for the extract was 114.12 μg/mL; while the value for vincristine sulfate was 1.63 μg/mL. The extract's in vivo anticancer activity against EAC cell line was also remarkable. The 400 mg/kg of body weight dose of methanolic leaf extract showed tumor weight and EAC cell number values of 3.20 ± 0.20 g and 8.40 ± 0.51 cells/mL; both of these values were lower than the values obtained from the standard drug 5-fluorouracil.

## 1. Introduction

Gas chromatography mass spectrometry analysis is conducted on plant extracts to detect and measure volatile and semivolatile organic compounds. By comparing the plant's chemical profile with an existing library of compounds, this method provides a comprehensive chemical profile of the plant. It aids in the identification of plant components and understanding of the biological functions of the compounds obtained from plants [1, 2]. According to Young and Woodside (2001), the development of numerous chronic and degenerative diseases has been linked to oxidative stress. According to Halliwell (1994), a large variety of plants contain chemicals with antioxidant potentials [3]. A group of malignant disorders collectively referred to as cancer continue to be a serious global health issue. Different levels of control are involved in the etiology of cancer. Genetic and epigenetic alterations play key role in the conversion of normal cells to cancerous ones. Furthermore, it is recognized that, in the development of tumors by inducing mutations in DNA, immune system evasion, the tumor microenvironment, metastasis, and angiogenesis, reactive oxygen species (ROS) are crucial. One of the primary issues with cancer cells is that they can avoid dying because of unknown mutations, which leads to an accumulation of cells that then spread to different areas of the body [4]. It is commonly known that plants can provide clinically relevant anticancer chemicals. Examples of some plant derived chemotherapeutic drugs include flavopiridol for colorectal cancer, vinca alkaloids for leukemia, and paclitaxel for breast cancer [5]. Various methods are used to choose plants that might have novel biologically active chemotherapeutic substances [6, 7]. One method that is employed is the ethnomedical data approach, wherein the choice of a plant is predicated on existing knowledge regarding the plant's traditional medicinal applications [8]. As herbal medicinal plants possess antioxidant, cytotoxic, and anticancer properties, the current study focused on *Annona reticulata* Linn. The genus *Annona* contains over 100 species, and *Annona reticulata* Linn. is a member of the *Annonaceae* family. Numerous illnesses have been treated ethnomedically with the plant which include fever, ulcers, worm infestations, bacterial infections, hemorrhage, dysuria, intestinal disorders, and epilepsy. People of the Philippines, India, and a few other countries have implemented the plant's therapeutic potentials to cure various illnesses [9]. The 5rSo, investigation of the antioxidant, cytotoxic, and anticancer potentials of this ethnomedically important herb, *Annona reticulata* Linn., was conducted in this study.

## 2. Materials and Methods

### 2.1. Preparation of Plant Extract

The plant part (leaves) of *Annona reticulata* Linn. was taken from Savar, Dhaka. After being sun-dried separately, the plant material (leaves) was dried at a lower temperature (no more than 50°C) in Gallenkamp air oven (Size 1) until it was ready for grinding. Subsequently, a high-capacity grinding mill was used to pulverize the plant components into coarse powders. Six hundred and thirty g of the prepared dried plant material were placed in dark-colored flasks, sealed with 12.6 L of methanol (solvent), and kept at room temperature. After a day, the infusions were filtered using Whatman No. 1 filter paper, and any remaining residue was extracted once again using the same amount of solvent. The procedure was repeated after 48 h. With a rotary evaporator set to 40°C, the combined extracts were dried off under vacuum. The completion of the extraction is indicated by the confirmation of depletion of the plant materials. After that, the plant material was dehydrated and immersed in 1 L of distilled water. For 7 days, the plant components were submerged in water in a sealed container with periodic shaking and stirring. Each extract was passed through a brand-new cotton bed filter. A sticky crude extract concentrate was produced by drying the filtrates at 40 ± 2°C. The extracted materials were kept in sterile sample containers and refrigerated at 4°C with appropriate labelling [10].

### 2.2. Phytochemistry

#### 2.2.1. GC-MS Analysis of the Methanol Extract From *Annona reticulata* Linn. Leaves

Using a Shimadzu GCMS-TQ8040, the GC-MS analysis was carried out. Helium was used as the carrier gas. Column oven temperature was adjusted to 50°C and gradually reached to 300°C with hold periods of 1, 2, and 7 min, respectively, according to the GC settings. With a sampling time of 1 min, a flow control mode set to pressure at 53.5 kPa. The total flow value was set at 11 mL/min and injection was performed in splitless mode at 250°C. Temperatures of interface and ion source were 250°C and 230°C under the MS conditions. In complex plant extracts, this setup offers great sensitivity and specificity for detecting and measuring volatile and semivolatile compounds through comparison with library compounds [11].

### 2.3. In Vitro Evaluation of Antioxidant Activity

#### 2.3.1. 2,2-Diphenyl-1-Picrylhydrazyl (DPPH) Radical Scavenging Assay

The stock solution is serially diluted to achieve the concentrations of 400 μg/mL, 200 μg/mL, 100 μg/mL, 50 μg/mLl, and 25 μg/mL. Extract solution (1 mL) and DPPH solution (2 mL) was taken together. Incubation (25°C) for 30 min in a dark place was carried out. Measurement of absorbance at 517 nm was then conducted. The percentage of scavenging activity and IC_50_ value were computed [12–14].

#### 2.3.2. Nitric Oxide (NO) Scavenging Assay

Test tubes were filled with test extract (4 mL) and ascorbic acid (standard) in varying strengths. Addition of sodium nitroprusside was carried out. Incubation (30°C) for 120 min was conducted. Then, the addition of Griess reagent was done. Absorbance was measured at 550 nm and compared with blank. Then, the calculation of percentage (%) of scavenging activity and IC_50_ values was done [15].

#### 2.3.3. Reducing Power Assay

The addition of 2.5 mL of a 1% solution of potassium ferricyanide was done after taking 2.0 mL of the plant extract or standard of various concentration solutions. After 10-min incubation period, each test tube received 2.5 mL of a 10% solution of trichloroacetic acid. Centrifugation at 3000 rpm of the entire mixture was conducted for 10 min. Two and half mL of distilled water was added after the removal of 2.5 mL of the supernatant solution. Half mL of ferric chloride solution (0.1%) was added to each test tube. Determination of the absorbances of the solutions was done at 700 nm with the aid of a spectrophotometer. At 700 nm, the absorbance of the blank solution was also measured. Increased absorbance of reaction mixture worked as indicator of higher reducing power.

#### 2.3.4. Determination of the Total Antioxidant Capacity

The reagent solution (3 mL) was added to the tube containing test extract (300 μL) or standard solutions of varying concentrations. The reaction was completed by incubating (95°C, 90 min) the test tubes. Measurement of absorbances at 695 nm was conducted in comparison to blank. After that, the antioxidant activity was computed and represented in ascorbic acid equivalents [16].

#### 2.3.5. Determination of the Total Phenolics Content

One mL of the plant extract (200 μg/mL) and gallic acid at varying concentrations were added to test tubes. Five mL of Folin–Ciocalteu reagent solution was diluted 10 times and added to the test tubes. The addition of 4 mL of sodium carbonate solution (7.5%) to the test tubes was done and the mixture was properly shaken. Incubation of extract solutions for 1 h (20°C) was carried out to complete the reaction. Then, the solutions' absorbance at 765 nm was measured by using a spectrophotometer in comparison to a blank [13, 17, 18]. The formula used by Pathak and Das, 2018, was employed to quantify total phenolic component in the extract and the result was represented as gallic acid equivalents (GAE) [19].

#### 2.3.6. Determination of the Total Flavonoids Content

One mL of the plant extract (200 μg/mL) or standard solution of varying concentrations was placed in test tubes. After that, 3 mL of chloroform, 200 μL of aluminum chloride solution (10%), 200 µL of potassium acetate solution, and 5.6 mL of purified water were subsequently added. Then, incubation for 30 min at room temperature was conducted. Next, the solution's absorbance at 415 nm was measured by utilizing a spectrophotometer in comparison to a blank [17, 18, 20]. Total amount of flavonoid components in plant extract in quercetin equivalents (QE) was determined by utilizing the standard formula.

### 2.4. In Vivo Evaluation of Cytotoxic Potential

#### 2.4.1. Brine Shrimp Lethality Bioassay for Assessing Cytotoxic Activity

To obtain a transparent sea water solution, 38 g of sea salt (devoid of iodine) were taken and added one litter of distilled water. After that, the mixture was filtered for the test organism, *Artemia salina* leach was utilized. The addition of seawater was done in little tank, and one end of the container was covered after the addition of shrimp eggs. For the shrimp to hatch and develop into nauplii, 2 days were allotted. The entire hatching period was spent with a steady supply of oxygen. Nauplii free of egg shells were collected from the illuminated area of the tank because hatchling shrimp are drawn to light (phototaxis). The removal of the nauplii was carried out with the use of a pipette. Then, to improve vision, dilution with fresh seawater was done. Each test sample weighed 32 mg. After weighing, 200 μL of DMSO was used to dissolve the sample and then added sea water to make the final volume of 20 mL. Consequently, 1600 μg/mL was the stock solution's concentration. Next, using sea water, the solution was serially diluted to 800, 400, 200, 100, 50, 25, 12.5, and 6.25 μg/mL. In this investigation, vincristine sulfate was employed. Vincristine was tested at extremely low concentrations (10, 5, 1, 0.5, 0.25, 0.125, and 0.06 μg/mL) due to its high cytotoxicity as an alkaloid. Sea water (4.95 mL with ten nauplii) was taken in three premarked test tubes and then 50 μL of DMSO was added (these tubes served as control groups). After 24 h, magnifying glass was used to examine the test tubes. Then, the surviving number of nauplii was calculated and percentage of mortality was then calculated for each concentration. Microsoft Excel was utilized for examining the data. LC_50_ value (which is computed via the linear regression approach) is typically utilized for demonstrating the effectiveness of plant products. However, the LC_90_ values for test fraction and the standard cytotoxic medication vincristine sulfate were also determined in a comparable manner [21–23].

### 2.5. In Vivo Evaluation

#### 2.5.1. Experimental Animals

This investigation employed Swiss albino mice weighing 25–30 g, which were collected from the department's animal house. In Jahangirnagar University Pharmacology Laboratory, methanol leaf extract of *Annona reticulata* Linn. was investigated pharmacologically. The animals were kept in regular laboratory conditions within polypropylene cages, which featured a proper dark-light cycle, relative humidity (RH 55 ± 5%), and a temperature of 25 ± 2°C. The mice had unlimited access to water and were fed pelletized mouse feed from ICDDR, B. Every action involving the handling of animals was conducted by obeying the guidelines provided by the committee for animal ethics of Jahangirnagar University (Ref no: BBEC, JU/M2024/11 (137)).

### 2.6. Assessment of In Vivo Anticancer Activity Against Ehrlich's Ascites Carcinoma (EAC) Cell Line in Mice

#### 2.6.1. Collection of Tumor Cells

EAC cells for transplantation were acquired from Jahangirnagar University in Savar, Dhaka.

#### 2.6.2. Transplantation of Tumor Cells

The first step was to render the malignant mice unconscious with ketamine hydrochloride. One mL syringe was then used to collect the ascitic fluid. It was then prepared for cell counting under an electron microscope by diluting it twice with 0.9% NaCl saline solution and then diluting it once more with phosphate buffer solution. The mice in each group were then given intraperitoneal injections of tumor cells. A total of 0.5 mL of treated ascitic fluid containing 2 × 10^6^ tumor cells were given intraperitoneally (i.p) to each mouse [24–26].

#### 2.6.3. Treatment Protocol

Four groups each of five mice were taken. Intraperitoneal injection of EAC cells was given to all the mice. This day of implantation was regarded as the first day. The following day, treatment began. The following treatments were administered to each group in due order: negative control (distilled water, orally), positive control (5-fluorouracil, 20 mg/kg, intraperitoneally), and two doses of methanol extract (200 and 400 mg/kg, orally). For the next 14 days, the treatment was continued. Mice's body weight was monitored carefully throughout the entire treatment period [25].

#### 2.6.4. Evaluation of the Anticancer Activity

Mice were sacrificed by cervical dislocation the next day, following an overnight fast. The retro-orbital route was used in the collection of blood, while the ascitic fluid was collected from the peritoneal cavity. A number of antitumor parameters were carefully recorded, in addition to some hematological and biochemical parameters [26, 27].

### 2.7. Anticancer Parameters

i. Tumor weight: mice were weighed both prior to and following the ascitic fluid collection in order to determine the tumor weight [26].
ii. Tumor cell count: hundred times' dilution of ascitic fluid was carried out with phosphate buffer solution and by utilizing white blood cells (WBC) pipette the diluted fluid was taken. After that, the placement of diluted cell suspension was done on Neubauer's counting chamber and the quantity of cells was calculated by the utilizing the following formula [28]:

(1) $$\begin{array}{c} \text{Cells}/\mu L=\left[\left(N_{c}\times D_{c}\right)÷\left(N_{s}\times V_{s}\right)\right]. \end{array}$$

Here, *N*_*c*_ = number of cells, *D*_*c*_ = correction for dilution, *N*_*s*_ = number of squares counted, and *V*_*s*_ = volume of one square.

#### 2.7.1. Hematological Parameters

The estimation of hemoglobin (Hb%), red blood cells (RBCs), WBC, and platelets was conducted on the blood that was collected from each mouse.

#### 2.7.2. Biochemical Parameters

Serum biochemical parameters such as aspartate aminotransferase (AST), bilirubin, alanine aminotransferase (ALT), and creatinine were measured according to the method described by Kumar et al. (2015) [29]. Commercially accessible kits were used for all the analysis.

### 2.8. Statistical Analysis

The observed data from in vivo studies was analyzed using SPSS 27.0. The findings of the experiment were represented using the mean ± SEM. The statistical significance was determined and compared with the control at different *p* value thresholds (^∗^*p* < 0.05, ^∗∗^*p* < 0.01, and ^∗∗∗^*p* < 0.001) using an analysis of variance (ANOVA).

## 3. Results and Discussion

### 3.1. GC-MS Analysis Report of the Methanol Extract of *Annona reticulata* Linn. Leaves

**Figure 1:**
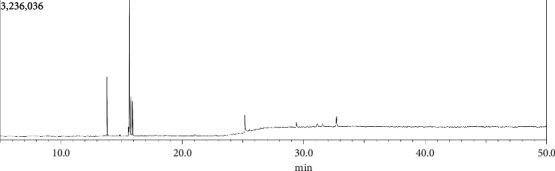
Total ionic chromatogram of *Annona reticulata* Linn. crude methanol extract.

The methanolic leaf extract of *Annona reticulata* Linn. contained 43 components, according to the library search report (Figure 1). The detected compounds are hexadecanoic acid, methyl ester; tridecanoic acid, 12-methyl-, methyl ester; tridecanoic acid, methyl ester; decanoic acid, methyl ester; methyl tetradecanoate; 9,12-octadecadienal; dichloroacetic acid, dodec-9-ynyl; E,E-1,9,17-docasatriene; 9-dodecyn-1-ol; 3-tetradecyne; 10-undecyn-1-ol; 13-tetradece-11-yn-1-ol; E-1,6-undecadiene; 11,14,17-eicosatrienoic acid, methyl ester; cis,cis,cis-7,10,13-hexadecatrienal; 5-pentadecen-7-yne, (Z)-; 3-heptadecen-5-yne, (Z)-; 1-tetradecen-3-yne; phytol; 3,4-dimethylcyclohexanol; 1,7-octadien-3-ol, 2,6-dimethyl-; oxirane, decyl-; methyl stearate; eicosanoic acid, methyl ester; octadecanoic acid, 17-methyl-, methyl ester; methyl tetradecanoate; 13-docosenamide, (Z)-; 9-octadecenamide, (Z)-; 8-methyl-6-nonenamide; 3-isopropoxy-1,1,1,5,5,5-hexamethyl-3-(trimethyl); thymol, TMS derivative; 1,2-bis (trimethylsilyl) benzene; 1-isopropoxy-5-propyl-2,3-bis-trimethylsilyl-1; 2-(N,N′,N′-trimethylhydrazino)-1,3-benzothiazole; 1-hexadecanesulfonamide, N-(3-aminopropyl)-; 3,5-bis (trimethylsilyl) cyclohepta-2,4,6-trien-1-one; trisiloxane, 1,1,1,5,5,5-hexamethyl-3,3-bis (trimethylsilyl) oxy-; trimethylsilyl-di (timethylsiloxy)-silane; pterin-6-carboxylic acid; 1,4-bis (trimethylsilyl) benzene; cholesterol 3-O-[[2-acetoxy] ethyl]-; Androstane-11,17-dione, 3-[(trimethylsilyl) oxy; and 1,5,6,7-tetramethylbicyclo [3.2.0] hept-6-en-3-yl. Several researchers also performed GC-MS analysis of methanolic leaf extract of *Annona reticulata* Linn. and found almost similar compounds [30, 31]. Among which some compounds were found to possess good antioxidant, cytotoxic, and anticancer potentials, such as phytol and 3-tetradecane have antioxidant activity [32, 33]. Moreover, phytol and 8-methyl-6-nonenamide are reported to have cytotoxic properties [34, 35]. Furthermore, 5-pentadecen-7-yne, (z)-; phytol; thymol, TMS derivative; 1,2-bis (trimethylsilyl) benzene; cholesterol derivative; and Androstane-11,17-dione, 3-[(trimethylsilyl) oxy]-, 17-[O (phenylmethyl) oxime], (3α,5α) are reported to contain anticancer activity [36–43].

### 3.2. In Vivo Evaluation of Antioxidant Activity

#### 3.2.1. DPPH Radical Scavenging Assay

**Figure 2:**
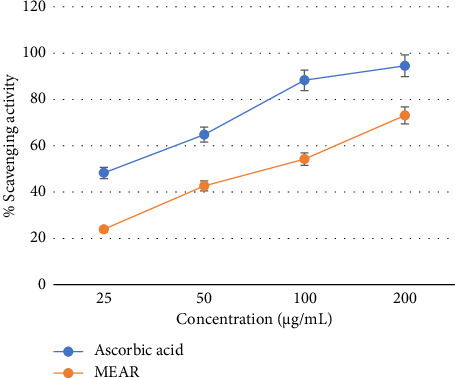
DPPH radical scavenging activity of the methanol extract of *Annona reticulata* Linn. leaves. Here, MEAR = methanol extract of *Annona reticulata* Linn.

Methanolic leaf extract from *Annona reticulate* Linn. demonstrated IC_50_ value of 86.93 µg/mL and ascorbic acid exhibited IC_50_ value of 18.62 μg/mL. As the concentration of methanolic extract increased, so does the degree of DPPH radical scavenging. The crude methanol extract of *Annona reticulata* Linn. exhibited dose-dependent DPPH radical scavenging, resembling the reference antioxidant ascorbic acid (Figure 2).

#### 3.2.2. NO Radical Scavenging Assay

**Figure 3:**
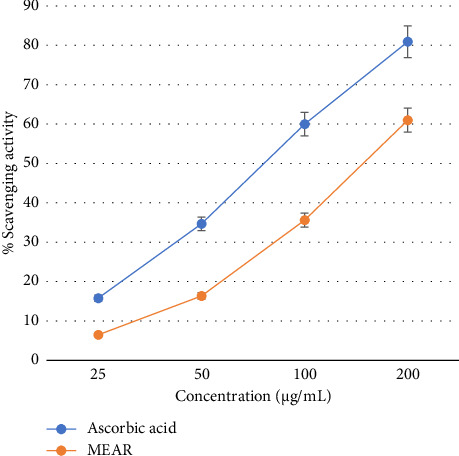
Nitric oxide (NO) radical scavenging activity of methanol extract of *Annona reticulata* Linn. leaves.

Methanolic leaf extract (IC_50_ value 148.42 μg/mL) demonstrated good scavenging when compared with ascorbic acid (IC_50_ value 76.31 µg/mL). NO scavenging potential was found to be dose dependent for both the test extract and standard (Figure 3).

#### 3.2.3. Reducing Power Assay

**Figure 4:**
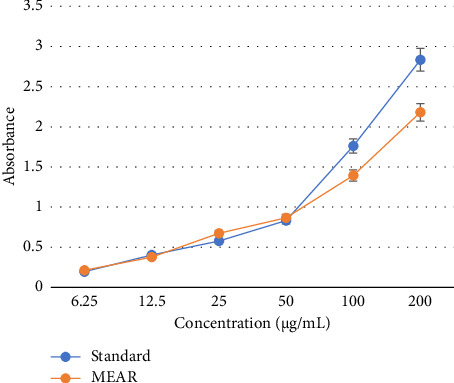
Reducing power of the methanol extract of *Annona reticulate* Linn. leaves.

The formation of Perl's Prussian blue color complex indicates toward the conversion of ferric to ferrous state, that is, an indicator to the extract's reducing activity. This reducing power of the extract is measured by using spectrophotometric analysis. Good reducing power was seen in the extract. The experiment found that the rise in concentration causes the rise in reducing power (Figure 4).

#### 3.2.4. Total Antioxidant Capacity

**Figure 5:**
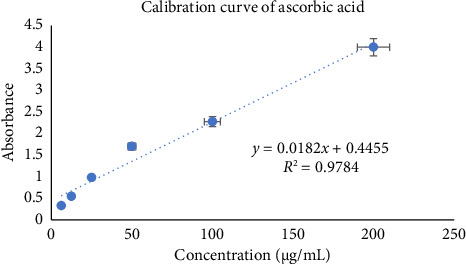
Calibration curve of ascorbic acid.

Using the phosphomolybdenum technique, calculation of the total antioxidant capacity of the test extract was done and represented in ascorbic acid equivalents. Determination of test sample's overall antioxidant capacity was conducted via the ascorbic acid standard curve (*y* = 0.0182*x* + 0.4455; *R*^2^ = 0.9784) (Figure 5). The obtained total antioxidant capacity value for methanolic leaf extract was 139.5 ± 7.21 mg/g ascorbic acid equivalents.

#### 3.2.5. Determination of the Total Phenolics Content

**Figure 6:**
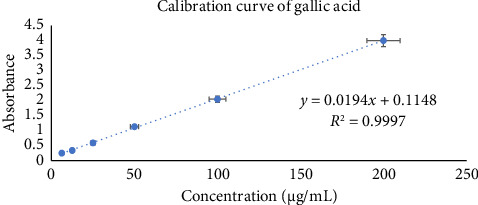
Calibration curve of gallic acid.

The Folin–Ciocalteu reagent was used to evaluate the total phenolic content of methanolic leaf extract of *Annona reticulata* Linn. The measurement was expressed GAE per gram of plant extract. Total phenolic contents of the plant extract were computed via the use of the Gallic acid standard curve (*y* = 0.0194*x* + 0.1148; *R*^2^ = 0.9997) (Figure 6). Phenols were discovered to be abundant in the methanol extract of *Annona reticulata* Linn. leaves, which is numerically 69.73 ± 0.26 mg/g GAE.

#### 3.2.6. Determination of the Total Flavonoid Content

**Figure 7:**
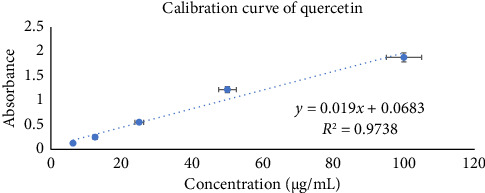
Calibration curve of quercetin.

The aluminum chloride colorimetric method was used to ascertain the amount of total flavonoid content in the methanol extract. The quercetin standard curve was utilized to obtain QE per gram of plant extract(*y* = 0.019*x* + 0.0683; *R*^2^ = 0.9738) (Figure 7) for the calculation of the total flavonoid content. The methanolic extract of *Annona reticulate* Linn. was found to contain high level of flavonoids, that is, 93.62 ± 0.15 mg/g QE.

Plant extracts' antioxidant activity has been assessed using a variety of methods. The involvement of free radicals in a wide range of clinical symptoms is well established. Antioxidants defend us against a variety of illnesses by combating free radicals. They work by preventing ROS from forming or by defending the antioxidant defenses. By utilizing DPPH radical purple-colored solution bleaching, one can assess the electron donating capacity of natural goods. By adding an antioxidant or radical species that decolorizes the DPPH solution, the technique scavenges DPPH. The strength and concentration of the antioxidants determine how much of a hue shift occurs. A substantial drop in the reaction mixture's absorbance suggests that the molecule being tested has strong free radical scavenging capabilities [44–46]. One of the main biological sources of ROS is thought to be the superoxide radical. Despite being a weak oxidant, the superoxide anion produces singlet oxygen and strong, harmful hydroxyl radicals, both of which exacerbate oxidative stress [47]. The test specimen's reducing power determines whether the test solution's yellow hue turns green in a reducing power assay. The Fe^3+^/ferricyanide complex is reduced to the ferrous form by the reductants present in the solution. Consequently, an absorbance measurement at 700 nm can be used to track Fe^2+^ [48]. Because of the scavenging properties of their hydroxyl groups, phenolic chemicals found in plants are very significant. Plant phenolic chemicals are classified into a number of groups, with flavonoids being the most prominent due to their strong antioxidant properties. Flavonoids, which are found naturally in plants, are believed to improve human health. Numerous antibacterial, antiviral, anti-inflammatory, anticancer, and antiallergic properties have been demonstrated in studies on flavonoidic derivatives. It has been demonstrated that flavonoids are very efficient scavengers of the majority of oxidizing molecules, including singlet oxygen, and other free radicals linked to a number of illnesses [49–51]. Physiologically significant chemicals of many different types can be synthesized by plants. ROS can be harmful to cells; however, cells can be protected from these detrimental effects by antioxidant chemicals that are found in many plants [52]. Antioxidant potential of the methanolic extract of *Annona reticulate* Linn. was validated using six previously reported techniques. As per the study's findings, the plant extract may contain phytochemical components that can scavenge possible harm by giving a free radical hydrogen. Furthermore, the findings of our investigation demonstrated that the test extract had a strong ability to scavenge superoxide radicals and associated with the total amount of flavonoids and phenols, indicating its potential as an antioxidant. Pathak and Das [19]; Jayaprakash; and Mondal et al. also confirmed the antioxidant potential of *Annona reticulata* Linn. in their studies [53, 54]. Furthermore, the GC-MS analytical report was used to identify the components found in the methanol extract. According to reports, phytol and 3-tetradecane, which are known to have antioxidant properties, were found in the methanol extract of *Annona reticulata* Linn. leaves [32, 33].

### 3.3. In Vivo Brine Shrimp Lethality Bioassay for Cytotoxic Activity

**Figure 8:**
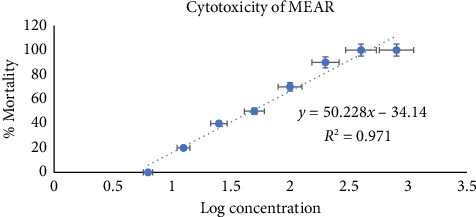
Graphical representation of the cytotoxic potential of methanol extract of *Annona reticulata* Linn. against brine shrimp nauplii.

Brine shrimp lethality bioassay was used to investigate the extract's potential cytotoxic effect. Anticancer medication vincristine sulfate displayed LC_50_ and LC_90_ values of 1.63 and 7.17 μg/mL in this investigation. By exhibiting LC_50_ and LC_90_ values of 114.12 and 502.85 μg/mL, the methanolic leaf extract was found to be extremely poisonous to shrimp nauplii (Figure 8).

Plant extracts that exhibit LC_50_ values less than 1000 μg/mL in this study are considered to be bioactive [55]. Methanol leaf extract's lethality to brine shrimp nauplii is displayed in Figure 8. It was discovered that the fatality exhibited by the extract was directly correlated with their concentration. The presence of cytotoxic principles in the extract was suggested by its concentration-dependent rise in the proportion of brine shrimp nauplii mortality. Moreover, certain cytotoxic components were detected from the findings of GC-MS analysis of the *Annona reticulata* Linn. methanol extract. The chemicals phytol and 8-methyl-6-nonenamide, which are both found in methanol extract, have cytotoxic properties [34, 35]. Many scientists confirmed the cytotoxic potential of various extracts obtained from *Annona reticulata* Linn. [56, 57].

### 3.4. Evaluation of In Vivo Anticancer Potential Against EAC Cell Line

In case of evaluating the anticancer activity of the extract, the changes in body weight of mice during the course of treatment is carefully monitored. In addition, the weight of tumor and the number of EAC cell in the ascitic fluid are calculated for each group to determine the anticancer potential of the plant extract [25–27]. From Table 1, it is clear that the obtained body weight change value was very alarming in the standard 5-FU-treated group. For the disease control group, the change in body weight was also notable. But for methanol extract–treated groups (both for 200 mg/kg and 400 mg/kg dosages), the outcomes for change in body weight were less than both disease control and standard groups; it is also notable that the body weight change value further decreases with increasing concentration of the test extract. The obtained tumor weight values were found to decrease in standard 5-FU and test extract–treated groups in comparison to the value obtained from the disease control group. In addition, it is noticeable that the tumor weight decreases with increasing dose of methanolic extract. The observed amount of EAC cell was also found to be lessened in standard and test extract treated groups; moreover, the observed amount of EAC cell was seen to be lowered with increase in the dose of methanolic leaf extract. Highest anticancer activity was obtained from 400 mg/kg body weight dosage of the test extract, which was greater than the activity gained from standard drug 5-FU. From above discussion, it can be said that the anticancer activity of the extract increases with increase in the dosage (Table 1).

During cancer treatment, generally the number of RBC, WBC, and platelet in cancerous animal changes as the homeostasis of the body disrupts badly. But with effective anticancer treatment, these changes can be minimized. For mice, the normal count of RBC, WBC, and platelet is (0.016–14.3) × 10^6^/cmm, (2–10) × 10^3^/cmm, and (14–1670) × 10^6^/cmm, respectively, and the normal range of Hb content in mice is 0.08–26.8 g/dL [58]. In this study, after dissection, blood was collected from the body of the test animals and was examined for determining the Hb content and the number of RBC, WBC, and platelet. From Table 2, it can be seen that the methanolic leaf extract at dosages of 200 and 400 mg/kg body weight were able to keep the values of Hb, RBC, WBC, and platelet within normal range, which is very good sign as many chemotherapeutic agents fail to perform this. Therefore, it can be said that the extract effectively maintained hematological parameters during the entire course of treatment.

During the treatment of cancer, liver and kidney can be affected as these organs are very crucial for the body's overall metabolism and excretion. As cancer alters normal physiological condition of human body, it can affect liver and kidney. For that reason, some important liver and kidney markers were estimated from the serum collected from the blood of animals treated with test sample to ascertain whether the sample can reduce the detrimental effects of cancer on these vital organs. The normal range of serum ALT, AST, bilirubin, and creatinine for mice is 17–77 IU/L, 54–298 IU/L, 8–33 mg/dL, and 0.2–0.9 mg/dL, respectively [59]. From Table 3, it can be found that the disease control group demonstrated alterations of kidney markers values with slightly higher liver markers values. While standard drug 5-FU also failed to maintain the creatinine level, the methanolic leaf extract at dosages of 200 and 400 mg/kg body weight maintained both liver and kidney markers levels effectively (only little fluctuation in bilirubin level for 200 mg/kg dosage). Therefore, it can be said that the extract has the potential to exert beneficial effect on liver and kidney during cancer treatment.

An extremely aggressive mammary adenocarcinoma that develops spontaneously in mice is called EAC. They are characterized by 100% malignancy, undifferentiation, rapid multiplication, and a reduced life span. EAC is similar to human tumors because of these characteristics. Apart from its similarity to human tumors, EAC is extremely sensitive to chemotherapy, which may account for how researchers use it in chemotherapeutic investigations [60]. The extract showed good anticancer activity against EAC cell line in this investigation. From the GC-MS analysis report, several anticancer compounds were identified in the test extract. Methanolic extract was found to contain the following compounds: 5-pentadecen-7-yne, (z)-; phytol; thymol, TMS derivative; 1,2-bis (trimethylsilyl) benzene; cholesterol derivative; and Androstane-11,17-dione, 3-[(trimethylsilyl) oxy]-, 17-[O (phenylmethyl) oxime], (3α,5α). All of these compounds are reported to possess strong anticancer activity [36–43]. Several *Annona reticulate* Linn. extracts have been tested for anticancer property by some researchers [61–63]. However, this study assessed the anticancer potential of *Annona reticulate* Linn. extract against the EAC cell line for the first time.

## 4. Conclusion

The study emphasizes *Annona reticulata* Linn. methanolic leaf extract's strong pharmacological potential as a viable option for antioxidant, cytotoxic, and anticancer applications. The outcomes of the current study indicate that the methanol extract from the leaves of *Annona reticulate* Linn. has significant antioxidant, cytotoxic, and anticancer properties. However, isolation of the bioactive compounds from the extract and subsequent tests of those may help elucidating the mechanism of actions by which different bioactivities were achieved and may pave the way finding a distinct conclusion regarding the current outcomes.

## Figures and Tables

**Table 1:** Effect of standard and MEAR on the changes in body weight and tumor weight and no. of EAC cell in EAC-induced tumor in mice.

Group	Body weight change (g) (Mean ± SEM)	Tumor weight (g) (Mean ± SEM)	No. of EAC cell (Mean ± SEM)
Disease control	8.20 ± 0.66	8.72 ± 0.46	22.20 ± 2.08
Standard	16.20 ± 0.38^∗∗∗^	5.08 ± 0.35^∗∗∗^	9.20 ± 0.86^∗∗∗^
MEAR 200	5.20 ± 0.66^∗∗^	3.60 ± 0.25^∗∗∗^	8.20 ± 0.58^∗∗∗^
MEAR 400	2.80 ± 0.58^∗∗∗^	3.20 ± 0.20^∗∗∗^	8.40 ± 0.51^∗∗∗^

*Note:* Standard is 5-fluorouracil.

^∗^ *p* < 0.05, significant.

^∗∗^ *p* < 0.01, highly significant.

^∗∗∗^ *p* < 0.001, very highly significant against control.

**Table 2:** Effect of the standard and MEAR on the hematological parameters in EAC-induced tumor in mice.

Group	Hb (g/dL) (Mean ± SEM)	RBC (10^6^/cmm) (Mean ± SEM)	WBC (10^3^/cmm) (Mean ± SEM)	Platelet (10^6^/cmm) (Mean ± SEM)
Disease control	8 ± 0.5	3.6 ± 0.2	15.3 ± 0.6	131.2 ± 5.1
Standard	10.4 ± 0.3^∗^	2.3 ± 0.2^∗∗∗^	13.2 ± 0.5^∗^	88 ± 2.3^∗∗∗^
MEAR 200	11.6 ± 0.6^∗∗∗^	4.2 ± 0.1	10.1 ± 0.4^∗∗∗^	146.8 ± 6.2
MEAR 400	12.7 ± 0.8^∗∗∗^	4 ± 0.2	10.9 ± 0.5^∗∗∗^	154.6 ± 3.8^∗∗^

*Note:* Standard is 5-fluorouracil.

^∗^ *p* < 0.05, significant.

^∗∗^ *p* < 0.01, highly significant.

^∗∗∗^ *p* < 0.001, very highly significant against control.

**Table 3:** Effect of the standard and MEAR on the ALT, AST, bilirubin, and creatinine levels in EAC-provoked tumor bearing mice.

Group	ALT (IU/L) (Mean ± SEM)	AST (IU/L) (Mean ± SEM)	Bilirubin (mg/dL) (Mean ± SEM)	Creatinine (mg/dL) (Mean ± SEM)
Disease control	13.9 ± 1.4	135.6 ± 12.5	4.4 ± 0.7	1.9 ± 0.06
Standard	17.2 ± 1.5	90 ± 2.9^∗∗∗^	8 ± 0.4^∗∗∗^	1.3 ± 0.04^∗∗∗^
MEAR 200	20.7 ± 1.3^∗∗^	102.1 ± 3.6^∗∗^	7.5 ± 0.3^∗∗∗^	0.9 ± 0.09^∗∗∗^
MEAR 400	23.1 ± 1.1^∗∗∗^	97.4 ± 2.2^∗∗∗^	8.8 ± 0.2^∗∗∗^	0.9 ± 0.07^∗∗∗^

*Note:* Standard is 5-fluorouracil.

^∗^ *p* < 0.05, significant.

^∗∗^ *p* < 0.01, highly significant.

^∗∗∗^ *p* < 0.001, very highly significant against control.

## Data Availability

All data and materials are contained and described within the manuscript. The plant's materials for the study were identified and voucher specimens are deposited at Bangladesh National Herbarium DACB no. 114865.
